# Plant–microbe interactions ameliorate phosphate-mediated responses in the rhizosphere: a review

**DOI:** 10.3389/fpls.2023.1074279

**Published:** 2023-06-09

**Authors:** Sakineh Abbasi

**Affiliations:** Department of Microbiology, Faculty of Biological Sciences, Alzahra University, Tehran, Iran

**Keywords:** phosphate solubilizing bacteria, drought, microbiome, plant response, biostimulant

## Abstract

Phosphorus (P) is one of the essential minerals for many biochemical and physiological responses in all biota, especially in plants. P deficiency negatively affects plant performance such as root growth and metabolism and plant yield. Mutualistic interactions with the rhizosphere microbiome can assist plants in accessing the available P in soil and its uptake. Here, we provide a comprehensive overview of plant–microbe interactions that facilitate P uptake by the plant. We focus on the role of soil biodiversity in improved P uptake by the plant, especially under drought conditions. P-dependent responses are regulated by phosphate starvation response (PSR). PSR not only modulates the plant responses to P deficiency in abiotic stresses but also activates valuable soil microbes which provide accessible P. The drought-tolerant P-solubilizing bacteria are appropriate for P mobilization, which would be an eco-friendly manner to promote plant growth and tolerance, especially in extreme environments. This review summarizes plant–microbe interactions that improve P uptake by the plant and brings important insights into the ways to improve P cycling in arid and semi-arid ecosystems.

## The importance of P for the plant

Climate change has led to a higher aridity around the globe, which has negative effects on nutrient availability and plant growth. Additionally, the increasing demand for food as a result of our growing global population is another factor that brings nutrient limitations (Cho et al., 2021). The use of chemical nutrients in most terrestrial ecosystems improves plant production, but it has a negative environmental impact such as reducing the biodiversity in soil (Smith and Schindler, 2009; Capek et al., 2018; Wu et al., 2022b). The complex molecular and biochemical interactions between plants and soil biodiversity ultimately determine the composition of the root microbiome in response to different types of stress (Hartman and Tringe, 2019; Bender and van der Heijden, 2015). Phosphorus (P) deficiency is one of the factors that may affect these interactions.

P is a restricted and non-substitutable natural element in agricultural and forestry production. It has regulatory impacts on the growth and survival of both the host plant and the associated microbiome.

P solubilization can mitigate the negative effects of drought by improving plant tolerance, as P significantly increases root biomass and hydraulic conductance of the root under drought stress (Tariq et al., 2017). Higher root biomass improves the ability of roots to absorb water in soil, contributing to drought tolerance. The strategies for improving plant-available P can positively affect soil biodiversity, which can improve plant resilience to other biotic and abiotic stresses. Additionally, the promotion of plant–microbe interactions for P supply can improve soil aggregation in arid areas (Rillig and Mummey, 2006; Smith and Schindler, 2009).

Here, we review some adapted microbial interactions that scavenge accessible P from the soil for plant uptake. We also present some positive microbial interactions that ultimately help to shape the root microbiome leading to improved P availability in the soil. Finally, we describe the multifunctional effects of P as a bio-stimulant on plant growth promotion and induced tolerance under stress conditions.

## Plant molecular adaptations for P uptake

Drylands form 41%–45% of the land area, which is occupied by 31% of the world’s human population (Metcalf et al., 2021). It has been shown that P deficiency restricts plant growth, especially in arid ecosystems, and P cycling is determined by both geochemical and biological factors. The amount of water, soil type, P concentration, and plant diversity can be considered as aridity indices of desert environments (Metcalf et al., 2021). Plants in desert conditions have evolved several morphological, metabolic, and physiological modifications, enabling the P acquisition from the dust (up to 30%). In P-poor desert ecosystems, plants absorb P *via* roots and leaves from the deposited dust particles during desert storms (Gross et al., 2021). In addition, P limitation and root properties can explain local plant species diversity (Lamont, 1981). For instance, non-mycorrhizal dependent plants such as Proteaceae are dominant (Lamont, 1981) and their growth in these environments is linked to their short-specialized roots that obtain P by expressing carboxylates (Lambers et al., 2006).

**Figure 1:**
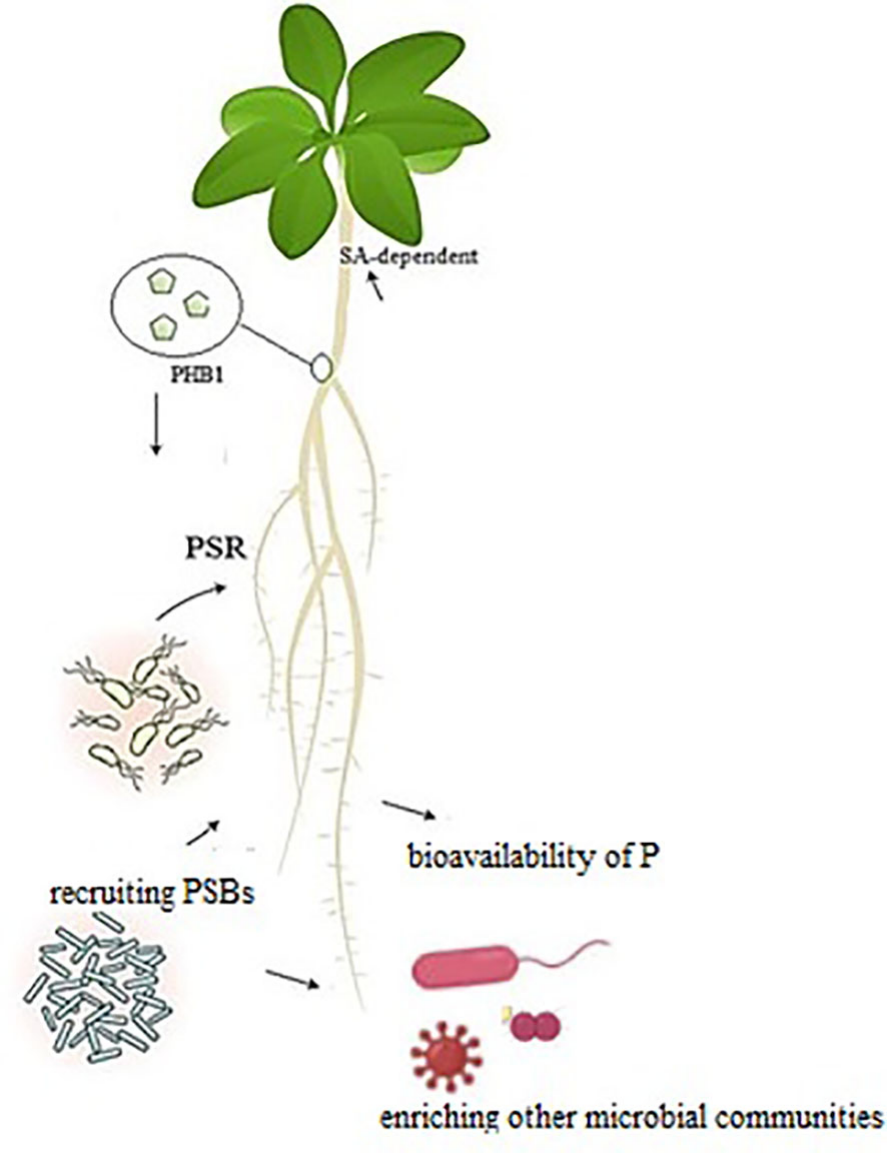
P-deficiency activates phosphate starvation response (PSR), regulated by the transcription factor *PHR1* to adjust plant growth and metabolic activity accordingly. *PHR1* and PSR regulate plant responses to abiotic stress, which is crucial for plants. Phosphate solubilization bacteria by enriching soil biodiversity leads to increased plant tolerance to encounter biotic and abiotic stresses. The SA-responsive genes are activated along with PSR.

The other strategy that plants have adapted to cope with P deficiency is the activation of phosphate starvation response (PSR) coordinated by *PHR* genes to regulate plant growth and metabolic content (Isidra-Arellano et al., 2021). *PHR1* gene and the PSR not only modulate the plant responses to P deficiency in abiotic stresses but also interact with valuable soil microbes that provide soluble P (**Figure 1**). Accordingly, plant nutrition and immunity are increased by harnessing beneficial microbial interactions. In *Arabidopsis thaliana*, *PHR1* can bind to the promoter of alkalinization factor (RALF) genes and induce the expression of these genes under P starvation (Tang et al., 2022). The RALF genes conjugated with a recognition receptor kinase FERONIA trigger pathogen-associated molecular pattern-triggered immunity to specific plant pathogens. Suppression of the plant immune system via PHR1-RALF-FERONIA permits the colonization of roots by the specific microbiome and alleviates P starvation through upregulating PSR genes (Tang et al., 2022). P deficiency or sufficiency triggers a different impact (positive/negative) to various biotic factors (beneficial microbes or pathogens) over a different PSR-PHR1 response. This also involves crosstalk among defense hormones, transcription factors (TFs), and P transporters (Pazhamala and Giri, 2023). Under normal P conditions, inositol polyphosphates recruit specific proteins to bind PHR1, in which case PSR genes are not expressed. The plants obtain P by constitutive mechanisms that induce defense responses to avoid pathogen infection. In P-poor conditions, PHR1 is linked to P1BS elements, which activates PSR genes or suppresses specific defense-related genes. The soil microbial community seems to be crucial for this response. As a consequence of the PSR expression, plants develop a smaller root system with more lateral branches and root hairs, stimulate P transporters, produce phosphatases to solubilize plant-unavailable P, and recruit AMFs or PSBs for extra utilization of P. Because of suppressed immune responses, these plants are more exposed to root colonization by soil microbes with a reduced resistance toward pathogens.

Phytohormones including salicylic acid (SA) or jasmonic acid (JA) play a vital role in microbe-mediated nutrient acquisition by different plants (Chan et al., 2021). Induction of JA-responsive genes is required during PSR but SA is a possible hub for P starvation-induced immunity (Castrillo et al., 2017). The changes in SA levels and SA-related signaling pathway have been observed under nutrient deficiency, especially under P deficiency and plant immunity.

## Impact of some abiotic inducers on soil P dynamics

### Drought

Drought stress drives strong effects on the soil P content (−9.18%). It was reported that drought treatments that included drying–rewetting cycles had no effect on P uptake by plants, while long-term drought stress reduced the P content (He and Dijkstra, 2014). The drought-induced P changes can be explained by the soil pH. Reducing soil pH leads to the solubilization of P into calcium phosphate under drought conditions (Zhang et al., 2020). In light of growing concerns about water and nutrient deficiency in terrestrial ecosystems, especially those used for agriculture, the number of studies has increased on the impacts of drought stress on the structure and function of the root concerning the improved plant tolerance (He and Dijkstra, 2014; Naylor and Coleman-Derr, 2018). The root microbiome under drought stress drives variations in root phenotypic traits and exudates (He and Dijkstra, 2014).

### Nitrogen (N)- to-P ratio

Plants have evolved morphological and physiological strategies for P acquisition such as N fixation in legumes under low-P conditions (Lazali and Drevon, 2021). When water is available to the plant; desert wild plant species *Glycyrrhiza uralensis* can reduce N-to-P ratio in soil by increasing N and P absorption. N inputs to the soil enhance N- to-P ratio, reduce N resorption, and increase P resorption by *G. uralensis*. On the other hand, P inputs to the soil reduce N-to-P ratio but increase root biomass (Huang et al., 2018; Srivastava et al., 2022). Dai et al. (2020) revealed that long-term P inputs to the soil enhance microbe-mediated P mobilization, but long-term N inputs reduce the efficiency of P-solubilization by reducing soil pH.

### Rhizosphere nutrients and root exudates

Nutrient availability in the rhizosphere affects plant growth depending on the soil properties and plant features (Capek et al., 2018). Some reports confirm the role of root exudates in plant–microbe interactions under P deficiency (Carvalhais et al., 2013; Liu et al., 2022). The highest rhizosphere nutrient accumulation observed in poor soil conditions confirms the negative correlation between soil chemical parameters and available P. The main nutrients needed for plants are more available in the rhizosphere compared to bulk soil (Liu et al., 2022).

## Impact of biotic factors on plant P uptake

### The role of fungi in P uptake

Plants rely on their interactions with microorganisms to scavenge the accessible P in the soil. The different microorganisms apply numerous strategies to obtain P from dryland soils for plant stabilization (Crain et al., 2018). Most terrestrial plants (except for Brassicaceae) are colonized with Arbuscular mycorrhiza fungi (AMF) and depend on the association between them, resulting in P uptake from soil (Soudzilovskaia et al., 2015). The efficacy of AMF in root colonization depends on the interactions between the root and rhizosphere microbiome (Reichert et al., 2022). The percentage of AMF colonization is nutrient-dependent, which is improved in low-P conditions but reduced in high P conditions possibly due to energy costs (Chen et al., 2020). The roots that efficiently acquire P were poorly protected against soil-borne fungal pathogens such as *Phytophthora.* By contrast, species with thicker, denser, and less branched fine roots that strongly depend on AMF for P acquisition are supposedly less susceptible to pathogens (Laliberte et al., 2015). Edlinger et al. (2022) reported that AMFs in grassland soils were much more effective in P uptake than in cropland soils, which were reduced by fungicide inputs. Thus, reducing the use of fungicides could lead to AMF enrichment and a better P uptake in agroecosystems (Edlinger et al., 2022).

Almario et al. (2017) evaluated the plant–fungi interactions in a non-mycorrhizal association under P-poor conditions that confirmed the role of root fungal populations in P acquisition. Comparative analysis of root and rhizospheric fungal communities of *Arabis alpina* under normal and P-poor conditions was conducted using amplicon sequencing. A taxon belonging to Helotiales was established with higher relative abundance in the roots of *A. alpina*, which was correlated with a higher P uptake and plant growth promotion. This fungus displayed mycorrhiza-like performances (AMF-like fungi), comprising the colonization of the root, endophytic lifestyle, and P transfer to the plant (Almario et al., 2017).

In addition, P-solubilizing fungi can increase plant growth by promoting nutrient cycling, the synthesis of plant hormones, enzymes, and volatile organic compounds (Vassileva et al., 2022).

### The role of bacteria in P uptake

P-solubilizing bacteria (PSB) can improve P acquisition efficiency of roots through P mineralization in available forms. PSBs from the rhizospheres of different plant species showed different solubilization efficiency, which highlighted that host plants can play roles in engineering their associated microbiome to improve P acquisition (Amy et al., 2022; Angulo et al., 2022). There is a growing interest in applying PSBs (Abbasi et al., 2019; Abbasi et al., 2020a; Abbasi et al., 2021a; Abbasi et al., 2021b) because of their advantages such as eco-friendliness, low production cost, and high efficiency (Timofeeva et al., 2022), especially for acidic soils where Fe ions can fix P (Cui et al., 2022). Cui et al. (2022) demonstrated that Fe–P mobilization by an endophytic *Streptomyces* sp. CoT10 strain in *Camellia oleifera* was positively correlated with siderophore production. Applying *Streptomyces* CoT10 strain in *C. oleifera* was shown to facilitate P and Fe absorption, improve P content in the soil, and promote plant growth. Moreover, inoculation of *Streptomyces* CoT10 strain enriched some dominant groups of Actinobacteria, Proteobacteria, and Firmicutes in the rhizosphere and balanced the microbial P- and Fe-related functions. Thus, siderophore-producing strains can increase beneficial microbial interactions for P mobilization in Fe-rich soils, which can have benefits for sustainable agriculture. Abbasi et al. (2020b) reported that co-inoculation of P-solubilizing and siderophore-producing *Streptomyces* strains not only promotes a higher plant growth but also induces resistance against a fungus-like pathogen *P. capsici* by regulating ethylene-dependent genes. In addition, Liu et al. (2023) reported that herbivores grazing in grasslands can enhance soil-accessible P content and alkaline phosphatase activity in the rhizosphere by enriching the abundance of some PSBs. Several drought-tolerant PSBs were recommended to combat P deficiency (**Table 1**). The application of these strains *via* innovative formulations could represent a promising approach to stress management in sustainable agriculture (Elhaissoufi et al., 2022).

**Table 1:** The biodiversity of drought-tolerant P-solubilizing bacteria.

PSM	References
*Pseudomonas*	(Oteino et al., 2015)
*Bacillus*	(Prakash and Arora, 2019)
*Sanguibacter*	*(* Amy et al., 2022 *)*
*Streptomyces*	(Abbasi et al., 2020a)
*Anabaena*	(Kollmen and Strieth, 2022)
*Arthrobacter*	(Kour et al., 2019)
*Klebsiella*	(Kour et al., 2019)
*Burkholderia*	(Kour et al., 2019)
*Phyllobacterium*	(Breitkreuz et al., 2020)

Srivastava et al. (2022) investigated the genetic and functional diversity of polyphosphate-accumulating bacteria (PAB) in the rhizosphere soil of *A. thaliana* and depicted the contributions of *Pseudomonas* sp. in the P cycling. Interaction of PAB with *A. thaliana* can increase in response to salinity stress to promote plant growth through P solubilization, auxin, and siderophore production. In this case, the occurrence of *Pseudomonas* strains was recognized as a P accumulator in the soil. The authors showed that functional genes, i.e., polyphosphate kinase and exopolyphosphatase, have important roles in polyphosphate metabolism and bacterial growth (Srivastava et al., 2022). The positive interactions between *Pseudomonas* strains, P cycling, and plant growth show a potential of P. sp. to be used as plant growth-promoting bioproducts under P deficiency.

### Effects of other organisms

Macroorganisms such as earthworms or some free-living nematodes could modulate changes in the microbial interactions (Wang et al., 2022a), which may affect nutrient cycling and P uptake. Wan and Wong (2004) reported that earthworms *Eisenia fetida* and *Pheretima guillelmi* can accelerate the availability of P. Additionally, Zheng et al. (2022) showed that some predatory nematodes such as bacterial-feeding *Eucephalobus* induce changes in the abundance of specific bacterial communities, such as alkaline phosphomonoesterase (ALP)-producing bacteria, and improve P uptake (Zheng et al., 2022; Wang et al., 2022b). These studies provide novel insights into microbial-mediated mechanisms stimulated by other soil organisms that can influence P cycling in soil and improve P uptake by the plant.

## Effect of P on stress management

### The roles of P in soil bioremediation

The plant–microbe associations are reasonably important in eco-friendly bioremediation by breakdown, adsorption, and volatilization of toxic compounds. These processes can transform toxic material into nontoxic or inactive compounds (dos Santos et al., 2021). The high levels of arsenic (As) in the soil and plant can cause health risks to humans due to its carcinogenicity. At the root surfaces, P significantly reduces As uptake and affects As biotransformation by competing for As transporters (Wu et al., 2022a). Indeed, concentrations can be reduced in plants *via* the manipulation of soil P content (Anawar et al., 2018; Wu et al., 2022b).

### The role of P in plant stress management

There are multiple examples of the importance of P in plant tolerance to abiotic and biotic stresses (Tariq et al., 2019; Mahreen et al., 2023). Mahreen et al. (2023) evaluated the effects of a consortium of drought-tolerant PSB strains from the rice rhizosphere (*Bacillus subtilis*, *B. cereus*, and *Brucella haematophilum*) on three rice genotypes. These strains significantly improved seedling growth and drought tolerance as compared to non-inoculated plants under drought-stress conditions. These responses were positively correlated with the accumulation of proline, relative water content, membrane stability, and production of antioxidants. RNA-seq analysis indicated that most of the transcriptional shifts were related to genes explained to the functional categories involved in P transport, organic acid secretion, hormone metabolism, and TF regulation. P can also play a role in plant defense against pathogens. For instance, Val-Torregrosa et al. (2022) showed that the increase of P content in *A. thaliana* can lead to an elevated resistance against fungal pathogens such as *Plectosphaerella cucumerina* and *Colletotrichum higginsianum*. The authors reported that overexpression of plant miRNAs such as MIR399, loss of function of PHOSPHATE2 (PHO2), and plant treatments with phosphates were responsible for an elevated P content and a subsequent plant resistance to these pathogens. However, this seems to be dependent on the plant and pathogen designation, as well as on the P content. For instance, another study showed that an excess of P in rice plants could lead to an increased plant susceptibility to the blast fungus *Magnaporthe oryzae* (Campos-Soriano et al., 2020).

## Conclusion

Plants rely on P for their growth and defense. However, a better understanding of the interactions among plants, microbiome, and P cycling in soil is needed to provide optimal conditions for P allocation to the plants, especially in drylands. The application of drought-tolerant P-solubilizing microbes has shown to be a promising strategy to alleviate biotic and abiotic stresses. In this review, we compiled the studies not only on the importance of P to the plant, but also on the strategies that plants use to access P in soils under limiting conditions such as drought. We discussed biotic and abiotic factors that affect plant-P dynamics and the importance of P for soil bioremediation and plant resistance to pathogens. Our special focus was on the microbial role in plant P uptake and on molecular mechanisms that drive these interactions. Further studies should focus on exploring the underlying P-mediated molecular mechanisms under drought, as well as the putative role of P-solubilizing bacteria in adaptation to extreme environments related to climate change. Conducting such studies could result in the improvement of plant tolerance to pathogens, decrease of agricultural costs of plant production, improvement of soil aggregation, and enhancement of P availability under limiting conditions in soil.

## Author contributions

The author confirms being the sole contributor of this work and has approved it for publication.
